# Surgical management of superior vena cava syndrome following pacemaker lead infection: a case report and review of the literature

**DOI:** 10.1186/1749-8090-9-107

**Published:** 2014-06-19

**Authors:** John Kokotsakis, Umar AR Chaudhry, Dimitris Tassopoulos, Leanne Harling, Hutan Ashrafian, Michail Vernandos, Meletis Kanakis, Thanos Athanasiou

**Affiliations:** 1 Cardiac Surgery Department, Evangelismos General Hospital, Athens, Greece; 2 Department of Surgery and Cancer, Imperial College London, 10th Floor, QEQM Building, St Mary’s Hospital Campus, South Wharf Road, London W2 1NY, UK

**Keywords:** Pacemaker lead, Superior vena cava syndrome, Cardiopulmonary bypass

## Abstract

Superior vena cava (SVC) syndrome is a known but rare complication of pacemaker lead implantation, accounting for approximately less than 0.5% of cases. Its pathophysiology is due to either infection or endothelial mechanical stress, causing inflammation and fibrosis leading to thrombosis, and therefore stenosis of the SVC. Due to the various risks including thrombo-embolic complications and the need to provide symptomatic relief, medical and surgical interventions are sought early. We present the case of a 48-year Caucasian male who presented with localised swelling and pain at the site of pacemaker implantation. Inflammatory markers were normal, but diagnostic imaging revealed three masses along the pacemaker lead passage. A surgical approach using cardiopulmonary bypass and circulatory arrest was used to remove the vegetations. Culture from the vegetations showed *Staphylococcus epidermidis*. The technique presented here allowed for safe and effective removal of both the thrombus and infected pacing leads, with excellent exposure and minimal post-procedure complications.

## Background

Superior Vena Cava (SVC) syndrome is an extremely rare but serious complication of pacemaker-lead implantation, characterised by symptomatic occlusion of the SVC [1,2]. Malignancy is considered to be the most common aetiology of SVC syndrome, but benign iatrogenic causes from intravascular devices (catheters, cardiac defibrillators and pacemaker wires) are becoming increasingly recognised [3]. Symptoms classically include neck, facial and/or upper limb swelling, along with other neurological complaints, which may all be exacerbated by different postures.

The frequency of pacemaker-induced SVC syndrome is difficult to ascertain; it can either be acute or chronic, with many patients being asymptomatic. The asymptomatic prevalence may be as high as 30% of all patients [4], with partial or complete venous obstruction. This is due to the development of an adequate venous collateral circulation. However, symptomatic cases of SVC obstruction from transvenous pacemaker implantation are more rare and are thought to account for less than 0.5% of all patients [5,6]. This can develop over a time period of between 2 days and 206 months [2]. The mortality associated with benign causes of SVC syndrome is low, however those patients who do become symptomatic are often debilitated by it, thus necessitating intervention.

The pathophysiology behind this phenomenon is likely endothelial disruption from mechanical stress or infection of the pacemaker leads, leading to inflammation and fibrosis and subsequently, thrombosis [7,8]. Several predictors may increase the propensity of thrombus formation and venous occlusion: upgrade of pacemaker devices; more than one pacemaker lead; device and/or lead infection; severed pacemaker leads [2,7,9].

The potential to develop SVC occlusion is a recognised indication for lead extraction. Other indications for device and lead extraction have been set out by the Heart Rhythm Society Expert Consensus [10]. These are broadly categorized into four main categories: a) infection including valvular endocarditis, lead endocarditis or sepsis; b) thrombosis or venous stasis including superior vena cava stenosis or occlusion with limiting symptoms; c) functional and d) non-functional leads [10,11]. With respect to pacemaker-related infections, the incidence varies greatly, with some reporting rates as low as 0.13%, but this can be as high as 19.9% [12]. *Staphylococcus* species account for the majority of infections (*Staphylococcus aureus* and *Staphylococcus epidermidis*), while other gram-negative bacilli, *Enterococcus faecalis*, *Pseudomonas aeruginosa*, *Candida*-species have also been implicated [12].

Management of pacemaker lead thrombosis can be either medical or surgical. Medical forms of management include anti-coagulation or thrombolysis, while surgical interventions rely on percutaneous endovascular intervention or lead extraction [13]. Lead extraction methods have evolved from transvenous procedures to laser-assisted extraction and in much more severe cases, the use of cardiopulmonary bypass. In this report and literature review, we present a case of SVC syndrome caused by pacemaker lead infection, which was managed with cardiopulmonary bypass and circulatory arrest.

## Case presentation

A 48 year-old Caucasian man attended outpatients’ clinic with a six-month history of localised swelling and pain at the site of his pacemaker implantation, particularly upon abduction of his right arm. He had twenty years previously undergone implantation of a VVI (ventricle; ventricle; inhibited) pacemaker due to sick sinus syndrome. Two years prior to his current attendance, the pacemaker was altered due to battery depletion and concomitantly upgraded to a DDD (dual-chamber; dual-chamber; dual triggered and inhibited) device.

**Figure 1:**
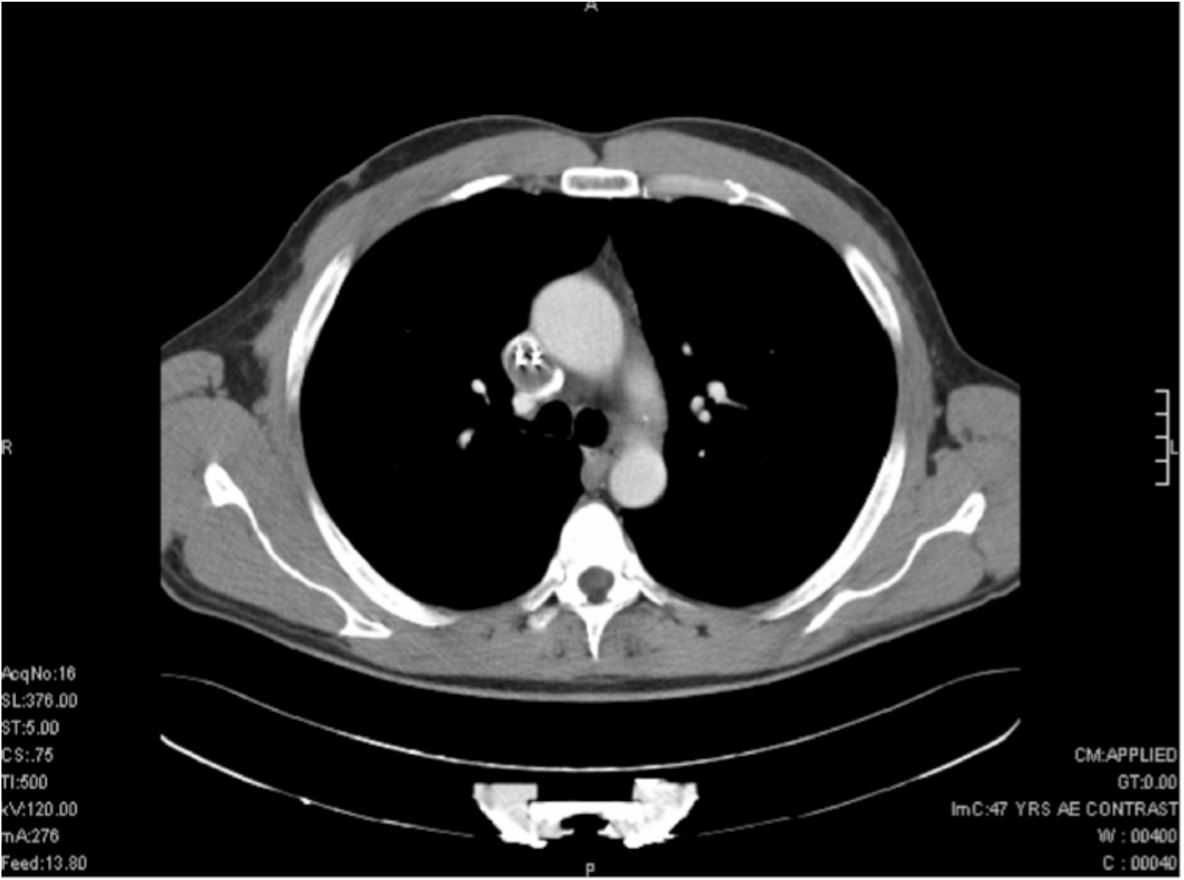
Transverse plane image of contrast-enhanced CT scan demonstrating two pacemaker leads within the SVC.

Physical examination revealed venous distension of the neck, upper chest and arms, but was otherwise normal. His electrocardiogram (ECG) was normal and he was not pacemaker dependent. Biochemical laboratory investigations including inflammatory markers were normal, and blood cultures were negative.A trans-esophageal echocardiography (TEE) identified three masses: a 40 × 18 mm mass at the atrial lead during its passage along the superior vena cava (SVC), which it occluded; a second 12 × 11 mm mass at the atrial surface of the ventricular lead; a third 5 × 5 mm mass during passage through the tricuspid valve. An open foramen ovale was further identified. Subsequently, contrast-enhanced chest computed tomography (CT) confirmed the TEE findings (Figure 1). On the basis of these findings a provisional diagnosis of concurrent SVC syndrome from infected vegetations of the pacing leads was made.

The presence of the large masses in the right heart cavities posed a risk of acute embolism, and so any attempt of transvenous retraction could not be made safely. It was therefore decided that the entire pacemaker system should be surgically explanted. The patient was transferred to the cardiac surgery department for an urgent surgical extraction of the leads and masses, as well as closure of the fossa ovale.

### Surgical procedure

**Figure 2:**
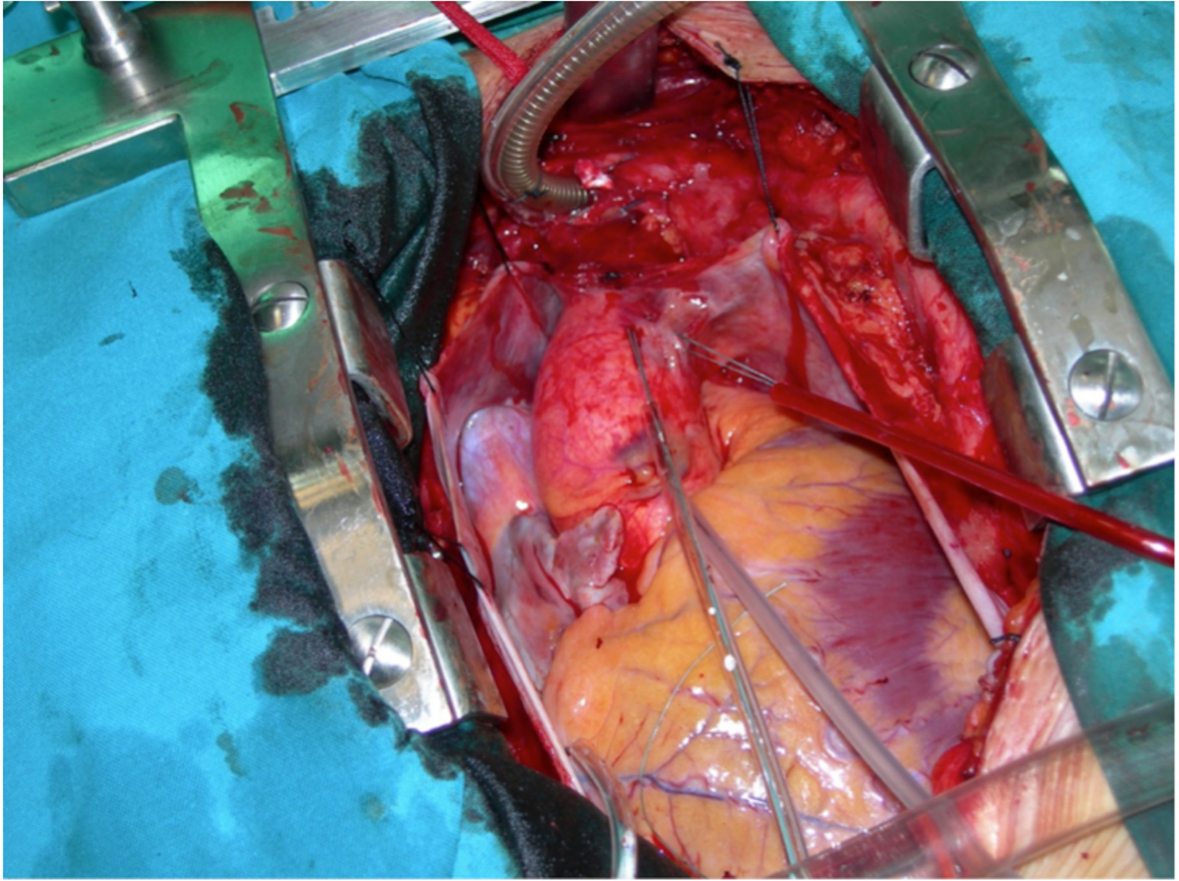
Cannulation of the innominate vein.

**Figure 3:**
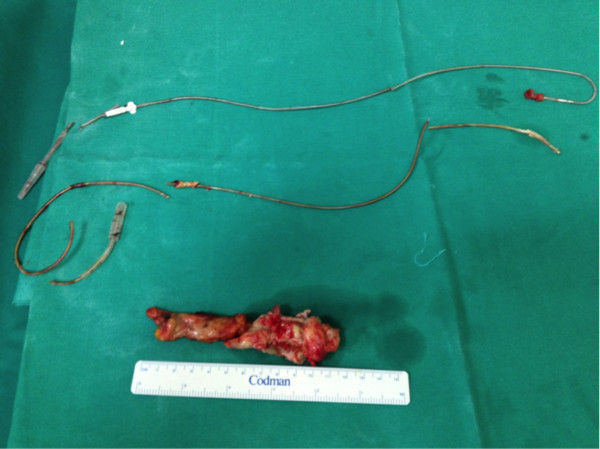
Extracted pacemaker leads.

**Figure 4:**
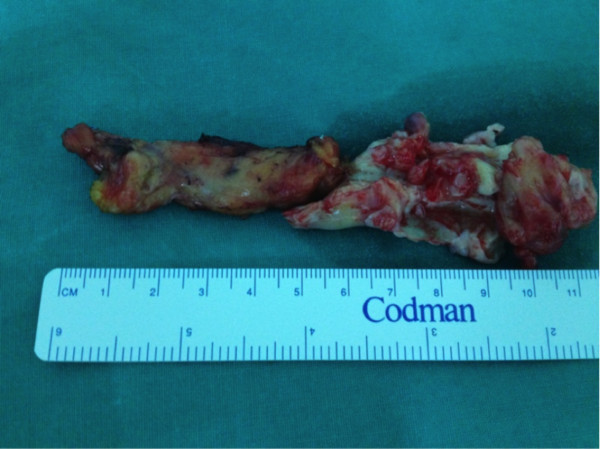
Extracted thrombus.

In the first instance the infected right infra-clavicular pacemaker pocket was opened, the generator was removed and endocardial leads were mobilized. A median sternotomy was then performed and the innominate vein, along with its confluence with the inferior vena cava (IVC) was dissected out. Heparin was administrated (300 μ/kg) and cardiopulmonary bypass (CPB) was established via the ascending aorta, innominate vein (Figure 2) and inferior vena cava (IVC). Myocardial protection was achieved with anterograde cold crystalloid cardioplegia (Custodiol 25 ml/kg) directly into the aortic root. The patient was cooled to a temperature of 26 degrees before commencing total circulatory arrest. The innominate vein and IVC were snared to isolate the right atrium and the thrombosed superior vena cava (SVC). The right atrium was opened and the incision was extended for 2 cm towards the right lateral wall of the SVC. The atrial and ventricular endocardial leads were carefully removed along with the adherent vegetations (Figures 3 and 4). The SVC was then completely thrombo-endartectomized. The patent foramen ovale was closed. The right atrial and SVC incision was closed with a running 4-0 polypropylene suture after de-airing the right side of the heart. CPB flow was re-instigated with a total circulatory arrest time of 38 minutes. Temporary epicardial pacing wires were placed in the right ventricle and right atrium. After a period of reperfusion and rewarming the patient was weaned from CPB. Wounds were closed after thorough antibiotic irrigation. The post-operative course was uncomplicated without the need for a further new pacemaker insertion.

**Figure 5:**
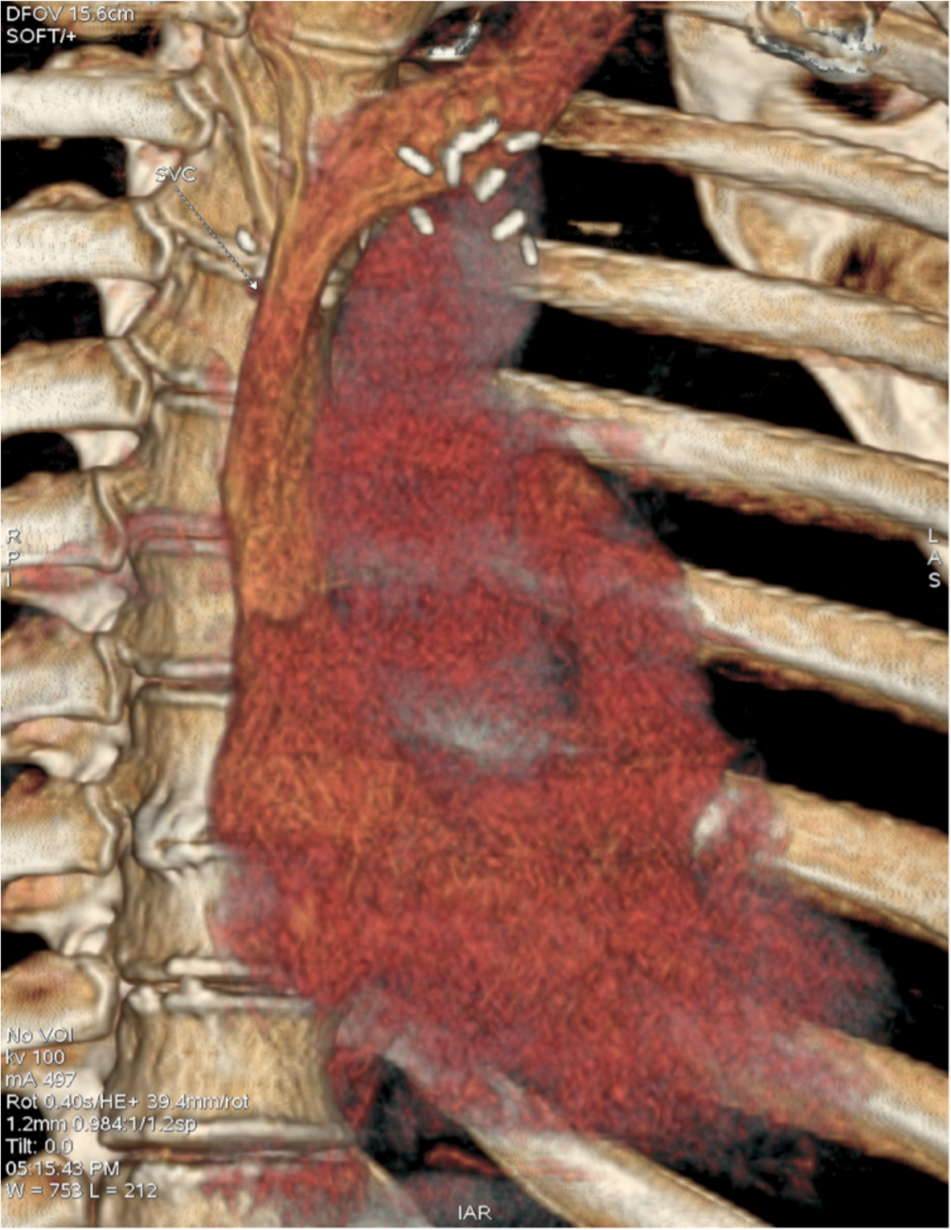
Post-operative 3-dimensional CT-imaging demonstrating complete clearance the SVC obstruction.

Histological examination of the second mass that occluded the SVC was characterised as a thrombus, whereas the other two masses were identified as vegetations. Culture from the vegetations and the pacemaker pocket yielded *Staphylococcus epidermidis*. This was treated with daptomycin 500 mg daily for six weeks. Post-operative cultures were negative, and post-operative CT showed a clear SVC (Figure 5). The patient was discharged after 7 days, with antibiotics for 6 weeks. Following the surgical procedure, there was substantial improvement in the patient’s clinical condition and the obstructive symptoms of the SVC syndrome subsided.

## Conclusions

We present an emergency case of SVC syndrome caused by transvenous pacemaker lead infection and thrombosis. A surgical approach, which required circulatory arrest and cardiopulmonary bypass, was required due to the extent of thrombus formation within the SVC. Because of the high risk of thrombo-embolic complications and because of symptom progression, prompt investigation and management was required.

Other surgical techniques including transveonous dilators, retrieval baskets and laser-assisted extraction have been previously described [14]. These percutaneous interventions are more frequently utilised. Byrd *et al*. report their experiences of intravascular extraction of 3,540 infected or problematic pacemaker leads, and demonstrate an overall major complication rate of 1.4%, with minor complications (including arrhythmias, pneumothorax, minor pericardial effusion) occurring in 2.3%. Importantly, they highlight greater risk with increasing number of leads and lesser experience [15]. Kennergren *et al*. described European experiences of laser-assisted extraction whereby there was extraction failure in 5.7%, and a total complication rate of 5.1% of the total 383 lead extractions [16]. Percutaneous stenting is another form of management for device-related SVC syndrome in order to retain the leads in situ and conserve its application with excellent short-term outcomes. Long-term data and efficacy for this modality, however, remains unknown. The choice between such procedures and a surgical approach remains unclear, but guidelines suggest that in those with larger masses, surgical extraction remains more desirable [10].

Our conventional approach of cardiopulmonary bypass and myocardial arrest allows both direct visualisation and complete removal of all pacemaker components (including leads) whilst avoiding dissemination of large thrombus. Wilhelm and colleagues present their experiences of 8 lead removals using extracorporeal circulation, and after a follow-up period of more than 18 months, 6 of 8 patients were alive and infection-free [17]. They also describe advantages of: less injury to heart structures; less vegetations from blood flow, which might otherwise cause mechanical stress; and the ability to perform concomitant procedures if required [17]. Okada *et al*. describe their recent experiences of 6 such procedures, with all surviving the follow-up period and one having a recurrence of infection [18]. Abad *et al*. demonstrate the well-tolerated use of cardiopulmonary bypass amongst seven patients with pacemaker infections; four with *Staphylococcus epidermidis*, two with *Staphylococcus aureus* and one *Pseudomonas aeruginosa*, with all being successfully managed with a follow-up period greater than two years and new epicardial devices inserted [19]. The use of cardiopulmonary bypass has been successfully described is several other reports, [20-23] with some reporting in the context of endocarditis or septicaemia, and one with further tricuspid valve debridement following endocarditis [23].

Our technique is novel in its cannulation of the innominate vein to complete the bypass circuit due to the extent of vegetations within the SVC. Previous practises have utilised the saphenous vein or the superficial femoral vein [9] whereas transfemoral and transiliac approaches have also been described [24]. One must however bear in mind both the general complications of CPB as well as specific complications including pericardial tamponade, haemothorax or pneumothorax, thrombo-embolism, peripheral lead escape or wound infection.

In terms of diagnosis, along with clinical suspicion, we used TEE to investigate the underlying cause for the patient’s symptoms. Ultrasonography for detection of severe innominate vein or SVC stenosis using sonography, pulse Doppler and colour flow have been shown to be accurate, and are useful for follow-up [25]. Contrast venography is considered to be the gold-standard for detecting venous obstruction but has drawbacks in terms of being invasive and inducing contrast nephropathy [26]. Spiral CT uses less contrast and benefits from excluding other causes of SVC obstruction [26]. Magnetic resonance imaging is also non-invasive but is a contra-indication in patients with pacemakers or implantable cardioverter defibrillator (ICD).

Our case has shown the safe approach for diagnosis and management of SVC syndrome caused by pacemaker lead thrombosis and infection. Although our approach of cardiopulmonary bypass and cardiac arrest is an invasive and technically challenging surgical procedure, it allows direct visualisation of the vegetations and the extent of disease. Our approach of innominate vein cannulation highlights a strategy for overcoming the problem of extensive disease within the SVC. Due to the heterogeneous nature of thrombus formation, medical or surgical diagnosis should be based upon clinical diagnosis and imaging. There is a lack of large multi-centre data to support one intervention over another. Future studies should therefore compare practices and provide long-term efficacies associated with cardiopulmonary bypass in the context of managing SVC syndrome from pacemaker lead infection and thrombosis.

## Consent

Written informed consent was obtained from the patient for publication of this case report and any accompanying images. A copy of the written consent is available for review by the Editor-in-Chief of this journal.

## Abbreviations

SVC: Superior vena cava; ECG: Electrocardiogram; TEE: Trans-esophageal echocardiogram; CT: Computed tomography; IVC: Inferior vena cava; CPB: Cardiopulmonary bypass; ICD: Implantable cardioverter defibrillator.

## Competing interests

There are no financial or non-financial competing interests.

## Authors’ contributions

JK, MK, DT, TA carried out the surgical procedure and participated in provision of clinical information and reviewed the manuscript. UC, HA, LH performed the literature review and drafted the manuscript. All authors read and approved the final manuscript.
